# Quantification of intracellular N-terminal β-actin arginylation

**DOI:** 10.1038/s41598-019-52848-5

**Published:** 2019-11-13

**Authors:** Li Chen, Anna Kashina

**Affiliations:** Department of Biomedical Sciences, University of Pennsylvania, Philadelphia, PA 19104 USA

**Keywords:** Cell migration, Post-translational modifications

## Abstract

Actin is a ubiquitous, essential, and highly abundant protein in all eukaryotic cells that performs key roles in contractility, adhesion, migration, and leading edge dynamics. The two non-muscle actins, β- and γ-, are ubiquitously present in every cell type and are nearly identical to each other at the amino acid level, but play distinct intracellular roles. The mechanisms regulating this distinction have been the focus of recent interest in the field. Work from our lab has previously shown that β-, but not γ-, actin undergoes N-terminal arginylation on Asp3. While functional evidence suggest that this arginylation may be important to actin’s function, progress in these studies so far has been hindered by difficulties in arginylated actin detection, precluding estimations of the abundance of arginylated actin in cells, and its occurrence in different tissues and cell types. The present study represents the first antibody-based quantification of the percentage of arginylated actin in migratory non-muscle cells under different physiological conditions, as well as in different cells and tissues. We find that while the steady-state level of arginylated actin is relatively low, it is consistently present *in vivo*, and is somewhat more prominent in migratory cells. Inhibition of N-terminal actin acetylation dramatically increases the intracellular actin arginylation level, suggesting that these two modifications may directly compete *in vivo*. These findings constitute an essential step in our understanding of actin regulation by arginylation, and in uncovering the dynamic interplay of actin’s N-terminal modifications *in vivo*.

## Introduction

Actin is a ubiquitous, essential, and highly abundant protein in all eukaryotic cells. Actin cytoskeleton function underlies muscle contraction, as well as cell adhesion, migration, and leading edge dynamics in many types of non-muscle cells. All these key physiological events critically depend on actin’s ability for rapid polymerization and disassembly, which are intricately regulated through a number of intracellular mechanisms that include modulation of activity of many actin-binding proteins, as well as direct modifications of actin itself.

In mammals, actin is represented by six highly similar isoforms, encoded by six genes differentially expressed in muscle and non-muscle cells^1^. The two non-muscle actins, β- and γ-, are ubiquitously present in every cell type and are nearly identical to each other at the amino acid level, except for 4 conserved substitutions in the very N-terminus. An increasing body of work suggests that despite this near-identity and ubiquitous co-expression in cells, β- and γ-actin play distinct intracellular roles^2,3^. The mechanisms regulating this distinction have been the focus of recent interest in the field.

Work from our lab has previously shown that beta, β- but not γ-actin undergoes N-terminal arginylation on residue 3 from the N-terminus (Asp)^4^, presumably following removal of N-terminal Met (previously shown to occur *in vivo* on the majority of de novo synthesized actin)^5,6^ and Asp2 (not previously demonstrated, but assumed based on mass spectrometry identification of the corresponding peptides in multiple actin samples). To date, N-terminal arginylation is the only known posttranslational modification that can selectively affect only one actin isoform. Our follow-up work demonstrated that the specificity of N-terminal arginylation to β-, but not γ-actin is not regulated at the amino acid level, but rather is driven by differences in their nucleotide coding sequences that confer differences in their translation rates^7^. β-actin arginylation has been suggested to be important for the maintenance of the cell leading edge^4^, as well as for defining the speed and directionality of cell migration^8^, and facilitating neurite outgrowth in neurons^9^. Moreover, recent studies showed that N-terminal actin arginylation, initially characterized only in mammalian cells, also exists in evolutionarily lower species: an abundant actin isoform in *Dictyostelum discoideum* undergoes N-terminal arginylation, and lack of arginylation in *Dictyostelium* leads to defects in actin cytoskeleton, substrate adhesion, and cell motility^10^. Thus, selective actin isoform regulation by N-terminal arginylation during cell migration may potentially turn out to be a universal mechanism conserved in different eukaryotic species.

Despite functional data about the importance of arginylation to the normal functioning of the actin cytoskeleton, progress in these studies so far has been hindered by the fact that detection of intracellular arginylated actin is very difficult and is not routinely observed during actin analysis. The majority of the intracellular actin is N-terminally acetylated, and the action of the recently identified actin N-terminal acetyltransferase NAA80 appears to be structurally incompatible with arginylation^5^. Thus, the questions of abundance and occurrence of N-terminal actin arginylation in different cells and tissues, and the exact percentage of actin arginylation among the total intracellular actin pool, constitute the focus of immediate importance in the field.

The present study represents the first quantification of the percentage of arginylated actin in different cells and tissues, under different physiological conditions. We find that while the steady-state level of arginylated actin is relatively low, it is consistently present *in vivo*, and undergoes a significant increase during cell stimulation and inhibition of N-terminal actin acetylation. We also find that arginylated actin is present in different mammalian tissues at different steady-state levels. Our current findings constitute an essential step in our understanding of actin regulation by arginylation, and in uncovering the dynamic interplay of actin’s N-terminal modifications *in vivo*.

## Materials and Methods

### Assurances

All methods were carried out in accordance with relevant guidelines and regulations. All experimental protocols were approved by University of Pennsylvania EHRS.

### Actin antibodies

The arginylated actin antibody used in this study was the rabbit polyclonal antibody raised by EMD Millipore, catalogue # ABT264, batch 15. This antibody was used at 1:2000 dilution in all Westerns and 1:200 dilution in the immunofluorescence images shown in the paper.

The acetylated β-actin antibody^11^ (Clone 4C2, EMD Millipore) is the mouse monoclonal antibody that displays very high specificity toward acetylated β-actin. It does not recognize any other actin species, including non-acetylated or arginylated actin. Thus, the β-actin signal described in this study actually refers to the acetylated β-actin, which is believed to equal ~99% of total intracellular actin that is not otherwise N-terminally processed^12^. This antibody was used at 1:2000 dilution for Westerns and 1:200 dilution in the immunofluorescence images shown in the paper.

### Cell culture

Immortalized wild type mouse embryonic fibroblasts (MEF) were cultured in DMEM (high glucose with GlutaMAX, Gibco Life Technology) with 10% fetal bovine serum and 1% Penicillin-Streptomycin (Antibiotic-Antimycotic solution; Life Technology) at 37 °C with 5% CO_2_. HAP1 (wild type and NAA80 knockout) were cultured as described in^5^.

### Western blot quantifications

Freshly collected tissues (brain, lungs, liver and kidney) from 3 different wild type mice were flash frozen and grounded in liquid nitrogen and weighed. MEFs were harvested by scraping and centrifugation. The pellets were resuspended in PBS, and spun down to remove the supernatant. Both tissue powder and cell pellets were lysed directly in 2× SDS sample buffer at the W:V ratio of 1: 10 (1 mg tissue: 10 µL buffer), followed by boiling the samples for 10 min. 10 µL of each sample was loaded for SDS-PAGE electrophoresis at 150 V. The gels were transferred to PVDF membrane at 100 V for 60 min. For loading control, the membranes after transfer prior to blocking were stained with LI-COR REVRT total protein stain as per manufacturer’s protocol and imaged using an LI-COR Odyssey® Infrared Imaging System at the 700 nm channel. The blots were then blocked and incubated with primary antibodies (1 to 2000 dilution) of rabbit anti-R-actin (ABT264 EMD Millipore) or mouse anti-β-actin (Clone 4C2, EMD Millipore) for 60 min at room temperature (~23 °C). Secondary antibodies (1 to 5000 dilution) conjugated to IRDye800 were used and images were acquired in the 800 nm channel using Odyssey Imaging System. Actin N-terminally arginylated on Asp3 (R-actin) heterologously expressed and purified from *Pichia pastoris*, and human platelet actin (85% β-actin, Cytoskeleton, Inc), were used as standards for R-and β-actin quantification respectively. For comparison of R-actin level in MEF cells with different confluency and treatment, the total protein intensity was used as internal loading control and the obtained signals were normalized to the first lane in each blot.

### Fractionation of intracellular actin and estimation of G- and F-Actin ratios

MEFs were harvested and lysed in F-actin stabilization buffer as previously described^13^ (50 mM PIPES, pH 6.9, 50 mM NaCl, 5 mM MgCl2, 5 mM EGTA, 5% glycerol, 0.1% NP40, 0.1% Triton X-100, 0.1% Tween 20, 0.1% 2-mercaptoethanol, 1 mM ATP, and protease inhibitor cocktail) followed by sequential centrifugation at 37 °C at 200 × g for 5 min (step 1), 1,500 × g for 15 min (step 2), 16,000 × g for 15 min (step 3), and 66,000 × g for 60 min (step 4). Supernatant from each step were measured by BCA protein assay, and mixed with 4% SDS buffer at 1:1 ratio. Pellets from each step were weighed out and lysed in 2% SDS buffer at 1 to 20 dilution (1 mg sample with 20 µL buffer) for the gel analysis. Supernatant and pellet fractions were analyzed by SDS-PAGE and Western blot as described above.

### Immunofluorescence staining

Cells cultured on coverslips were fixed by 4% paraformaldehyde (PFA) at 37 °C for 30 min, followed by washing with PBS three times, and then permeabilized for 5 minutes with cold methanol (−20 °C). Then the cells were washed with PBS three times followed by sequential incubation for 60 minutes in a blocking solution (5% goat serum in PBS), then with rabbit anti-R-actin (ABT264 EMD Millipore, 1:200) for 60 min at room temperature (~23 °C), and then with mouse anti-β-actin (Clone 4C2, EMD Millipore, 1:200) for 60 min at room temperature. Cells were washed with PBS for three times, then incubated for 1 hour at room temperature with Alexa Fluor 488-conjugated anti-mouse antibody and Alexa Fluor 555-conjugated anti-rabbit antibody (Life Technologies). After the staining, coverslips with cells were first incubated in PBS with DAPI for 5 min, then washed with PBS two times followed by mounting with ProLong Diamond anti-fade mounting media (Life Technologies). All images were acquired on a Nikon Ti microscope with Andor iXon Ultra 888 EMCCD camera and analyzed by MetaMorph software.

## Results and Discussion

**Figure 1:**
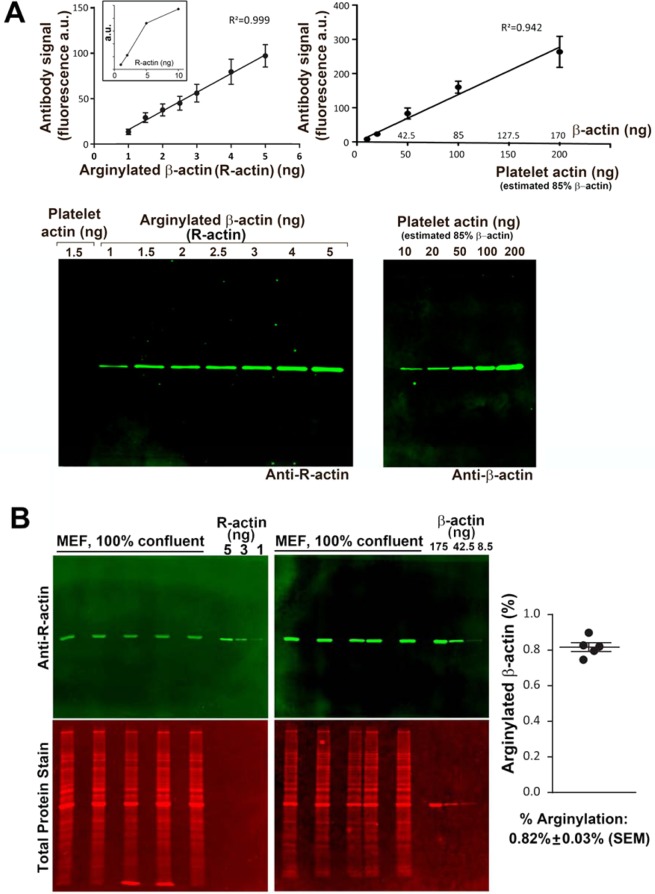
Quantification of the percentage of arginylated actin in mouse embryonic fibroblasts. (**A**) Calibration of the antibody signal using purified platelet actin (85% β-actin) and pure N-terminally arginylated β-actin (R-actin) expressed in *Pichia pastoris*. Error bars represent SEM, n = 6 for R-actin and 5 for platelet actin. The inset in the left graph shows the calibration curve with the additional point to show the limit of the linear range. (**B**) Quantification of the percentage of R-actin in confluent mouse embryonic fibroblasts (MEF). Error bars represent SEM, n = 5. See also Fig. S1 for antibody specificity and Fig. S2A for the raw data on the antibody signal used for the quantifications in this panel.

To quantify the percentage of intracellular N-terminally arginylated β-actin (R-actin), we used the commercially available R-actin and β-actin antibodies, as well as standard proteins, including fully arginylated β-actin produced in *Pichia pastoris*^14^, and human platelet actin (purchased from Cytoskeleton, Inc., 99% pure, containing 85% β-actin and 15% gamma non-muscle actin, according to the manufacturer’s data sheet). First, we probed these standard proteins at different concentrations by Western blotting with β-actin and R-actin antibodies, to determine the linear concentration range for the assay and confirm the antibody specificity (Fig. 1A). We also used standard peptides in dot blots to confirm the specificity of R-actin antibody to N-terminally arginylated β-actin sequence and lack of cross-reactivity with other naturally occurring N-terminal actin sequences (Fig. S1). Next, we prepared whole cell lysates from confluent cultures of immortalized mouse embryonic fibroblasts (MEFs) and used Western blotting and comparisons to the standard protein loads in the same gel to determine the amount of R-actin and β-actin in these samples (Fig. 1B). The signal from each antibody was converted to ng using the comparison to the protein standards loaded into the same gel (kept in the linear range, Fig. S2) to determine the ng amount of each protein in each lane. The ratio of these ng amounts of R-actin and β-actin in the same samples at the same load enabled us to quantify the fraction of the intracellular β-actin that is N-terminally arginylated (Fig. 1B, right). We found that in a confluent MEF culture, approximately 0.82% of the total β-actin is arginylated. This number is considerably lower than prior estimates^4^, but consistent with the fact that arginylated actin in cells tends to be difficult to detect.

**Figure 2:**
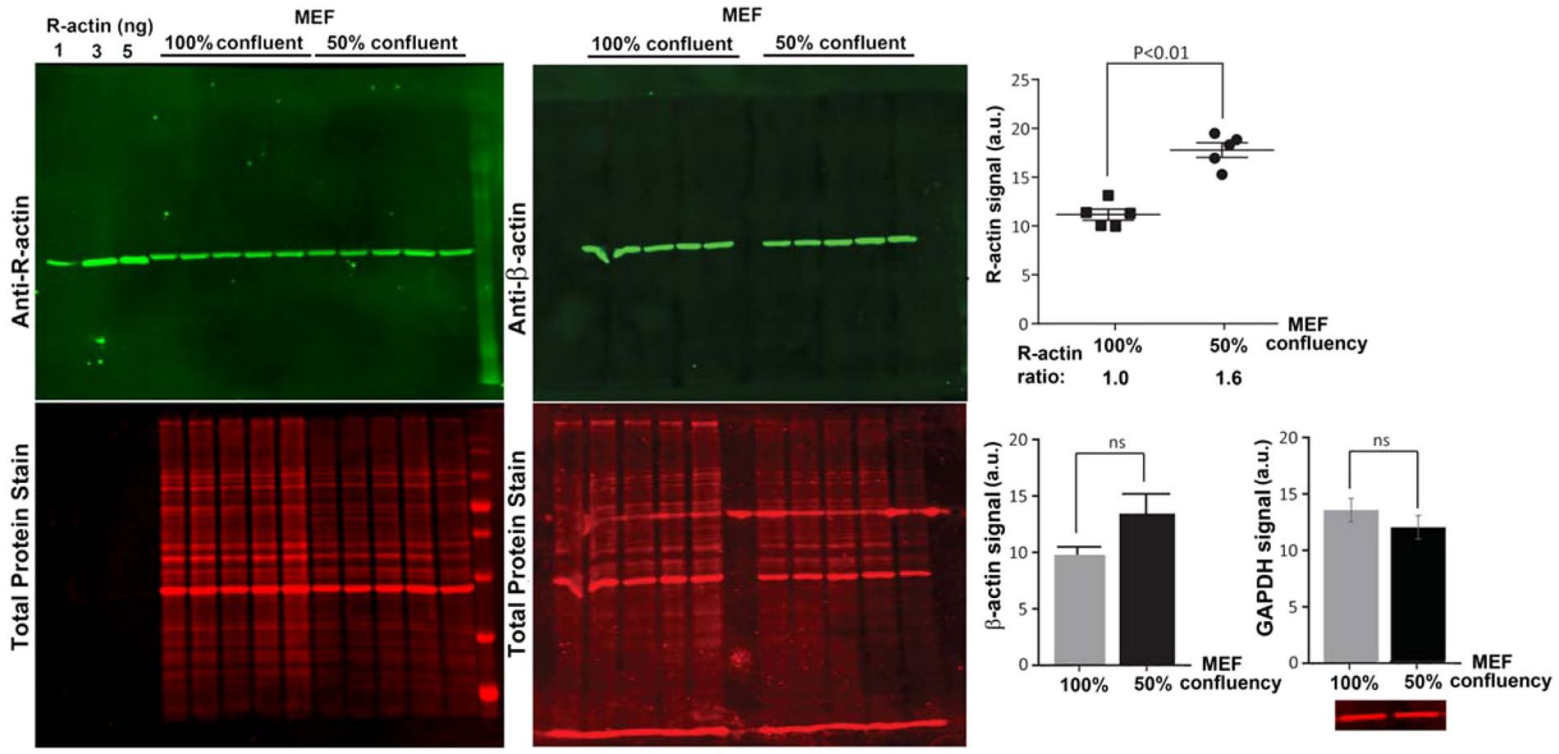
Percentage of actin arginylation partially depends on cell confluency. Left, Western blots of MEF cells with different confluency probed with antibodies against R-actin and β-actin as marked. Right top, quantification of total fluorescence from the R-actin band, normalized according to signal of total protein staining. Right bottom, quantification of β-actin and GAPDH in the same samples. Inset on the bottom right shows the representative GAPDH signal used for quantification. Error bars represent SEM, n = 5 for actin and 3 for GAPDH, Student’s t-test was used to determine the P value.

Our previous work showed that actin arginylation can decrease in cells undergoing serum starvation and recover after serum re-addition^8^, suggesting that this modification is dynamic and may be prevalent in migratory cells. The confluent cells used for the quantification above exist in contact-inhibited state that precludes directional migration, and thus are predicted to have lower arginylation level. To test whether less confluent cells (expected to be less contact-inhibited and more migration-enabled) have higher levels of actin arginylation, we compared R-actin signal in 50% confluent and 100% confluent MEFs, and found that 50% confluent cells have an approximately 1.6-fold increase in overall actin arginylation (Fig. 2). Control experiments showed no significant change in β-actin or GAPDH between these confluency states, however β-actin levels exhibited a decline with confluency which was below statistical significance (p-value > 0.1) (Fig. 2). Based on these results, we estimate that 50% confluent cells may contain up to 1.3% of arginylated β-actin, compared to the ~0.8% in 100 confluent cells (Fig. 2). It is possible that this percentage further increases at lower confluencies and/or upon additional cell stimulation.

**Figure 3:**
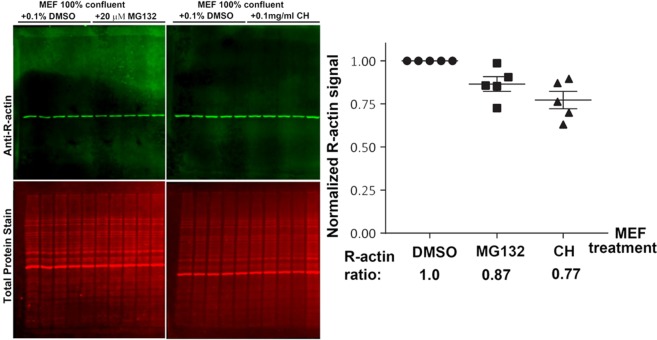
Percentage of N-terminal β-actin arginylation is not affected by inhibition of translation or proteasome degradation. Left, Western blots of confluent MEF cells treated with MG132, cycloheximide (CH), or 0.1% DMSO control f. Right, quantification of total fluorescence from the R-actin band, normalized according to the signal of total protein staining. Error bars represent SEM, n = 5.

It has been predicted by our previous work that β-actin arginylation may occur co-translationally, and thus may potentially depend on translation activity in the cells^7^. Additionally, a body of prior work links N-terminal arginylation to protein degradation via the proteasome, suggesting that R-actin *in vivo* may be at least somewhat less metabolically stable than non-arginylated actin^15^. To test whether the fraction of arginylated actin in the cell is sensitive to either translation or proteasome inhibitors, we compared the R-actin levels in cell lysates from confluent MEF cultures, treated with the translation inhibitor cycloheximide, proteasome inhibitor MG132, or DMSO control. Both inhibitor treatments led to a slight reduction in the arginylated actin levels compared to control, however these changes were not statistically significant (Fig. 3). Thus, translation and proteasome activity do not have a significant effect on the R-actin level.

**Figure 4:**
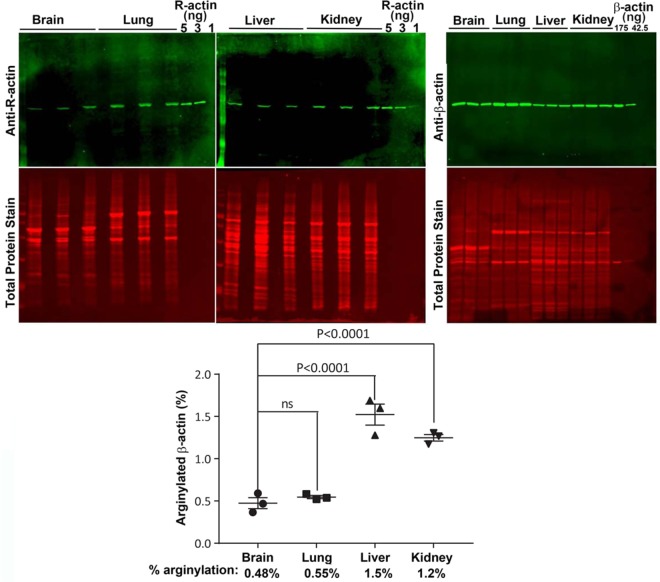
Different mouse tissues contain different levels of R-actin. Top, Western blots of four different tissues from wild type mice. Bottom, quantification of R-actin in different tissues. Analysis of variance (ANOVA) test was used to calculate the statistical difference between different tissues in the group. Error bars represent SEM, n = 3 biological replicates. See Fig. S2B for the raw data on the antibody signal used for the quantifications in this panel.

Multiple past studies demonstrate the role of arginylation in normal development and functioning of a number of organ systems, including brain, heart, and vasculature^16–20^. However, the presence of arginylated β-actin in any tissues or cell types besides MEFs^4^, neurons^9^, and glioblastoma cells^21^ has never been demonstrated before. To test for the presence and abundance of arginylated β-actin in different non-muscle tissues, we tested brain, lungs, kidneys, and liver derived from healthy wild type mice. All these tissues contained prominent levels of R-actin, suggesting that N-terminal β-actin arginylation normally occurs during their functioning *in vivo* (Fig. 4). Quantification of percentage of this arginylation in comparison to the β-actin levels in the same samples at the same load revealed the numbers comparable to MEFs – the lowest in the brain (0.48%), the highest in the liver (1.5%). Thus, actin arginylation occurs in different tissues and varies in levels between different cell types.

**Figure 5:**
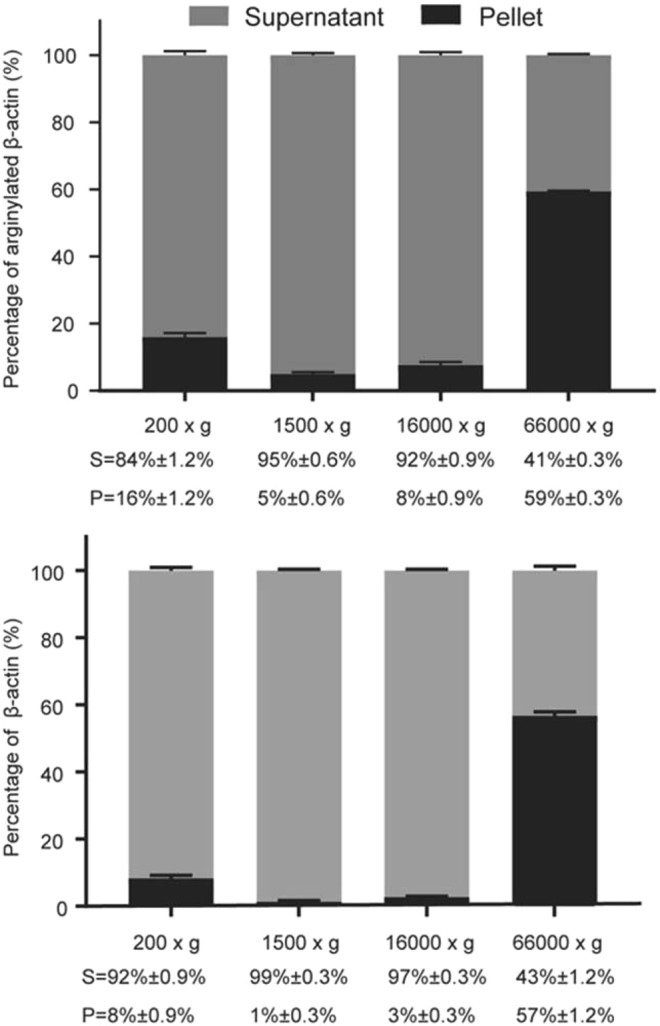
R-actin distribution between monomeric and polymeric actin fraction. Western blot quantification of R-actin and β-actin in subcellular fractions from MEF cell lysates. Bars show the percentages of R-actin and β-actin present in the supernatant (light gray) and pellet (dark gray) from different centrifugation steps. Error bars represent SEM, n = 3 independent fractionations.

We next used differential centrifugation of freshly prepared MEF lysates to examine the overall distribution of arginylated actin in different subcellular fractions, including its distribution between the F- and G-actin pool (Fig. 5). Notably, pellets from the initial centrifugation steps (200 g, 1,500 g, and 16,000 g, expected to contain the nuclei and large intracellular debris (200 g), as well as highly crosslinked and stable fractions of cytoskeleton (1,500 g and 16,000 g)) contained proportionally larger fraction of R-actin compared to the β-actin content in the same fractions (2–5 fold enrichment). In the final centrifugation step that pellets the majority of filamentous actin and separates it from the monomer pool, the distribution of R-actin and β-actin between the supernatant and pellet appeared similar (59% R- versus 57% β-actin). These results suggest that R-actin tends to be more associated with the polymeric and insoluble actin pool, and may exhibit a bias toward the nucleus and stably crosslinked larger cytoskeletal structures, potentially associated with the perinuclear cytoskeleton.

It has been previously found that in many migratory MEFs arginylated actin concentrates in a narrow zone along the cell leading edge^8^. We used immunofluorescence staining of a few selected cells where this distribution appears to be the most prominent, to crudely evaluate the maximum enrichment of R-actin at the leading edge. To do this, we measured the relative distribution of R-actin and β-actin signal intensity in these cells along the radial lines drawn from the nucleus into the center of the leading edge in selected images of cells showing the most prominent R-actin leading edge enrichment (Fig. S3). This relative distribution greatly varied from cell to cell, however we found prominent zones where R-actin distribution was markedly different from that of β-actin, suggesting that in these cells R-actin does not uniformly follow the intracellular β-actin pool. The highest R-actin: β-actin ratio determined from these scans in selected highly enriched zones was approximately 2:1 (Fig. S4). Area and intensity calculations suggest that in these extreme cases approximately 25% of the total intracellular R-actin is concentrated within the narrow leading edge zone, suggesting that the local enrichment of R-actin in the leading edge cytoskeleton may potentially be transiently high. While this estimate is qualitative and likely very variable between cells, it points to the possibility that local R-actin increase in different cell areas may exert localized effects on actin polymerization and protein binding.

**Figure 6:**
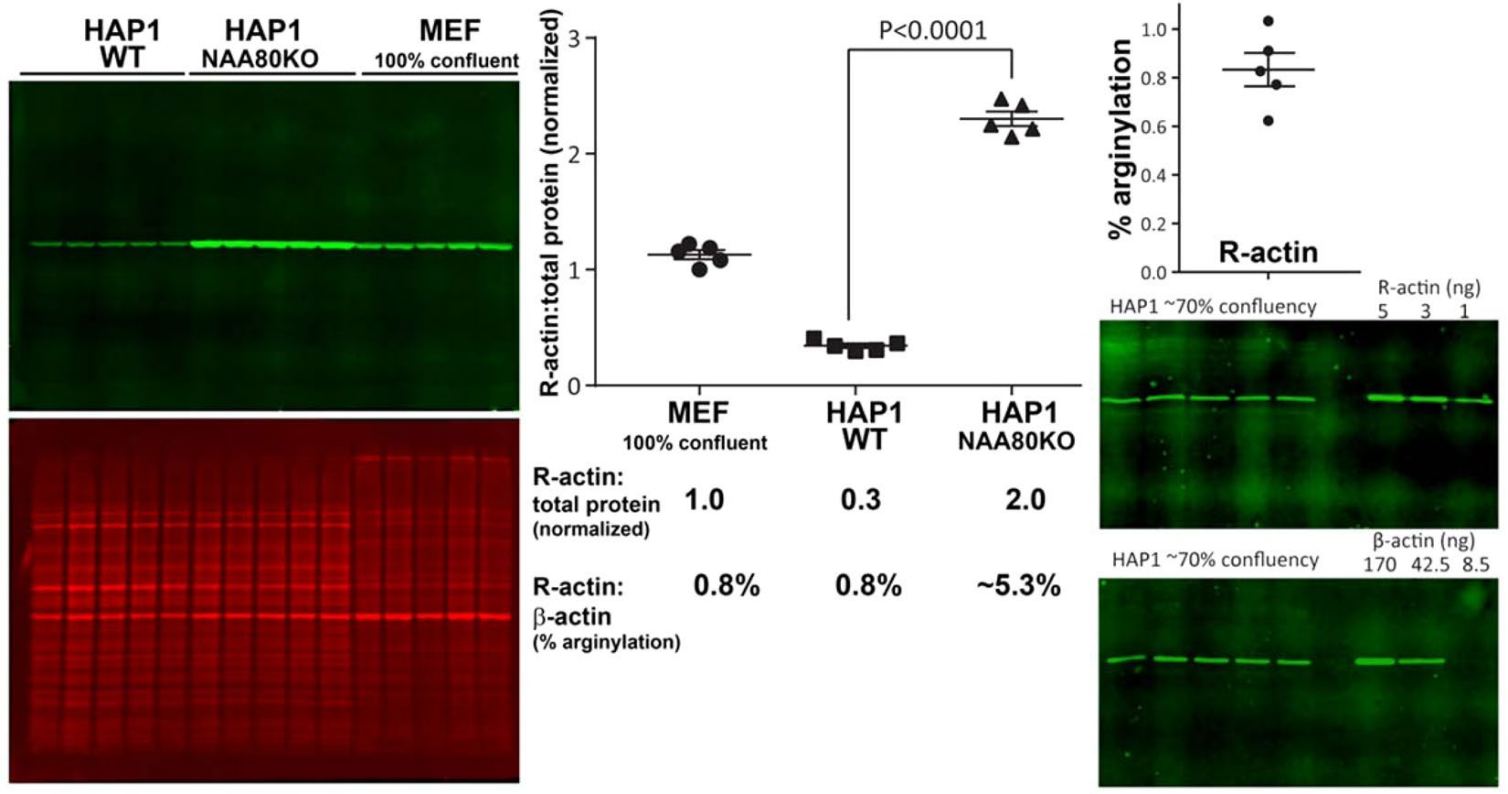
Lack of N-terminal actin acetylation in HAP1 cells leads to a dramatic increase in N-terminal β-actin arginylation level. Left, Western blots of HAP1 WT, HAP1 NAA80 knockout and 100% confluent MEF cells. Middle, quantification and comparison of the R-actin fluorescence signal from these different cell types. Signal of total protein stain was used as reference for R-actin normalization. The 5.3% arginylation in HAP1NAA80KO cells is shown as an estimate, since no β-actin signal can be detected in these cells. Right, quantification of the percentage of R-actin in wild type HAP1. Error bars represent SEM, n = 5, Student’s t-test was used to determine the P value.

The majority of intracellular actin is N-terminally acetylated^6^. Acetylation and arginylation are never found within the same actin’s N-terminal peptides, suggesting that these two modifications are likely mutually exclusive. To test whether inhibition of N-terminal actin acetylation has any effect on N-terminal actin arginylation levels, we compared the levels of R-actin in HAP1 cells (a human haploid cell line), either intact (wild type) or deficient in actin N-terminal acetyltransferase NAA80^5^. In HAP1 cells, which are less motile and very morphologically different from MEFs, actin constitutes a lower percentage of total intracellular protein, however arginylated actin percentage is consistent with the one measured in 100% confluent MEFs (approximately 0.8%, Fig. 6, right). Strikingly, deletion of NAA80 resulted in a nearly 7-fold increase in actin arginylation (Fig. 6, left and middle). In these HAP1NAA80 knockout cells, non-acetylated β-actin was no longer recognized by the β-actin antibody, thus precluding the possibility of measuring the percentage of β-actin arginylation, but extrapolating this fold change from the HAP1 wild type control, we estimate that approximately 5.3% of β-actin in these cells is arginylated. This level of arginylation approaches the level previously predicted by computational modeling to lead to significant effects on β-actin-dependent cell motility^4^. Notably, previously published data show that knockout of the arginylation enzyme ATE1 leads to a significant reduction in cell migration rate^4,19^, while NAA80 knockout leads to a substantial increase in the rate of cell migration^5^. Thus, it appears likely that these two modifications may exist in an interplay that could conceivably modulate the overall migratory behavior of non-muscle cells.

The quantification of N-terminally arginylated β-actin presented here constitutes an important first step in reconciling this enigmatic modification with the multitude of other actin modifications that exist in different cells and tissues. In our original paper identifying actin arginylation^4^, we used Coomassie-stained 2D gels from wild type and arginylation knockout MEFs to crudely estimate that up to 20% of intracellular actin (and up to 40% of β-actin) may be arginylated. In view of our current data, we now know that these approximations, based on a number of unverified assumptions, were grossly over-estimated. Our new result, showing that the actual steady-state level of N-terminally arginylated β-actin is under 1% of the total, is consistent with the fact that arginylated β-actin is very difficult to detect in cells, and to date only two groups have reported seeing it in their samples by mass spectrometry^4,21^. It is also consistent with prior observations that the majority of actin in many cell types is N-terminally acetylated. It is still possible, however, that in specific cell types and/or in different subcellular areas under specific physiological conditions N-terminal arginylation may affect a much higher fraction of actin.

Previous studies from our lab showed that deletion of arginyltransferase ATE1 leads to severe disorganization in actin cytoskeleton and impairments in cell migratory behavior^4,13^. In these studies, using complete ATE1-free background, it was impossible to evaluate which of the observed effects are due to the lack of actin arginylation itself and which are compounded by the impairments in other proteins that are also unable to undergo ATE1-dependent arginylation in this system. While transient transfection of GFP-fused arginylated actin construct into cells restores their leading edge morphology, this transfection does not fully rescue the cell spreading or other arginylation-dependent defects^4^. Thus, it is clear that effects of arginylation on cell behavior and actin cytoskeleton are complex, and likely extend beyond the N-terminus of actin.

Our earlier estimates of high percentage of actin arginylation in cells led to speculations that the effects of arginylation may  target large populations of intracellular actin by altering its global polymerization properties or interactions with its numerous binding partners. The present study, showing that the overall percentage of actin arginylation in most cells is very low, points to a different spectrum of possibilities. A low amount of arginylated actin is unlikely to exert a global effect on actin organization in the whole cell. It is possible, however, that all of this arginylated actin pool transiently concentrated in one spot may still lead to a powerful, potentially propagating, change in local actin organization. It is also possible that arginylation may make a fraction of actin uniquely suited for only a selected set of the its numerous functions. For example, actin arginylation may target it for transcriptional reprogramming in the nucleus, consistently with R-actin enrichment in the nucleus-containing fraction during subcellular fractionation seen in our study. Actin arginylation may also facilitate a select set of actin subunits to induce membrane binding at the cell leading edge, consistently with its preferential association with the detergent-labile zone at the cell’s leading edge^8^. Notably, a fraction of β-actin is locally translated at the cell periphery, and the exact role of this local translation, while important, is still not fully understood^22^. It is possible that this specific freshly synthesized β-actin fraction is targeted by arginyltransferase through its previously suggested association with the ribosomes^23^, thus pre-marking  a de novo synthesized actin pool for a locally specialized function in this particular subcellular zone. Since arginylation is incompatible with the action of NAA80 acetyltransferase that globally modifies the rest of the actin, such arginylation may be used to protect a small actin pool from N-terminal acetylation. Local differentiation of actin into the oppositely charged arginylated and acetylated pools can in principle exert strong effects on actin organization and dynamics.

## Supplementary information

Supplemental Figures and Legends
