# Supplementary material for: Quantification of intracellular N-terminal β-actin arginylation
Source: Sci Rep. 2019 Nov 13;9:16669. doi: 10.1038/s41598-019-52848-5 (PMC6853978; doi:10.1038/s41598-019-52848-5)
Supplement: Supplementary file 1 — Supplemental Figures and Legends [file 41598_2019_52848_MOESM1_ESM.pdf]

Quantification of intracellular N-terminal β-actin arginylation

Li Chen and Anna Kashina

Supplemental Online Information

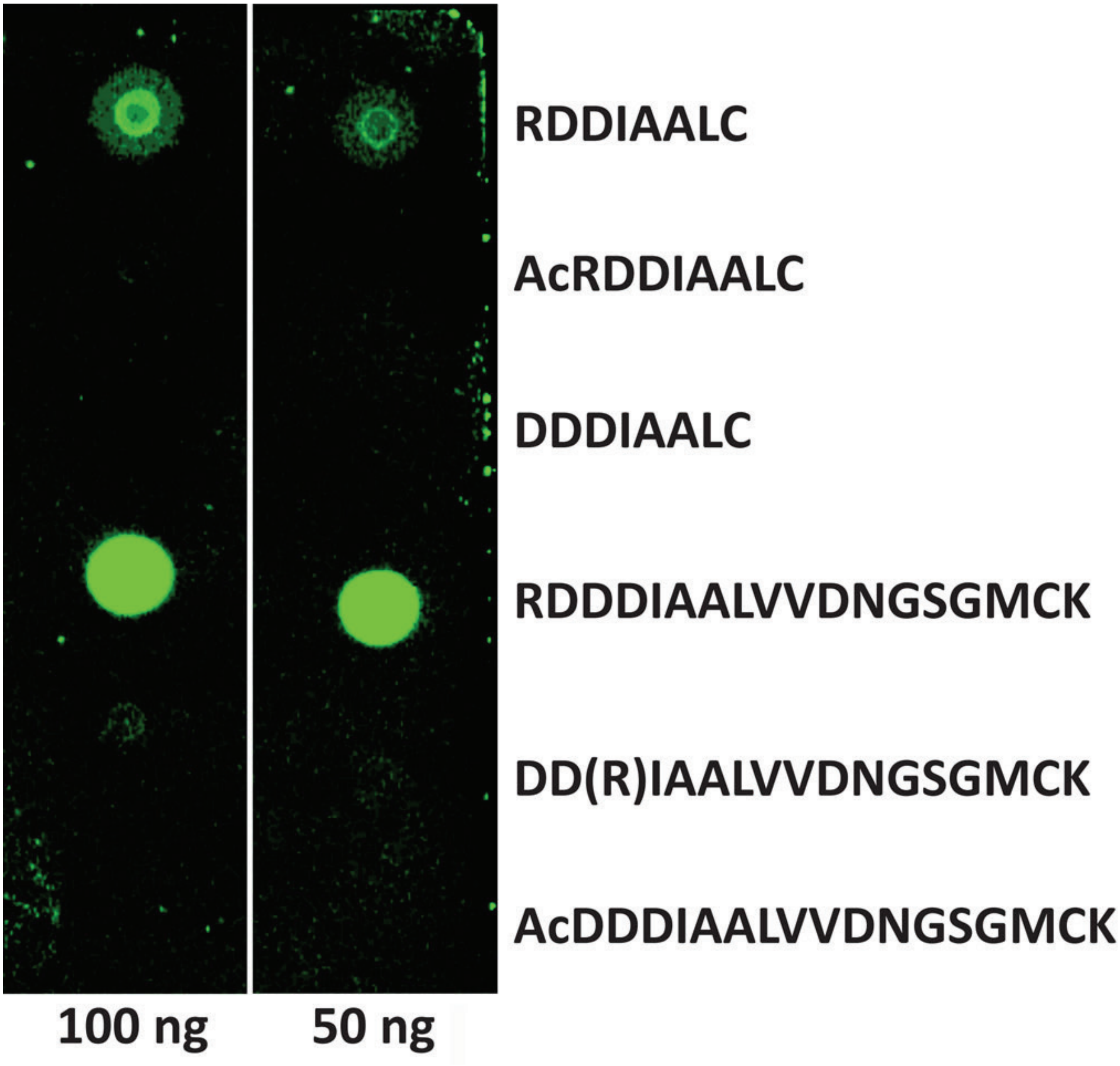

Figure S1. Antibody to R-actin is highly specific to the N-terminally arginylated actin peptide and does not recognize either N-terminally acetylated or non-acetylated pre-processed β-actin N-terminus. Western dot blots with R-actin antibodies against spots containing 100 or 50 ng each of synthetic peptides with the sequences indicated on the right. DD(R)… peptide was synthesized with a side chain addition of R to an N-terminal aspartic acid in the β-actin sequence. Longer β-actin peptide appears to have stronger antibody reactivity, potentially due to better presentation of the longer peptide on the nitrocellulose membrane.

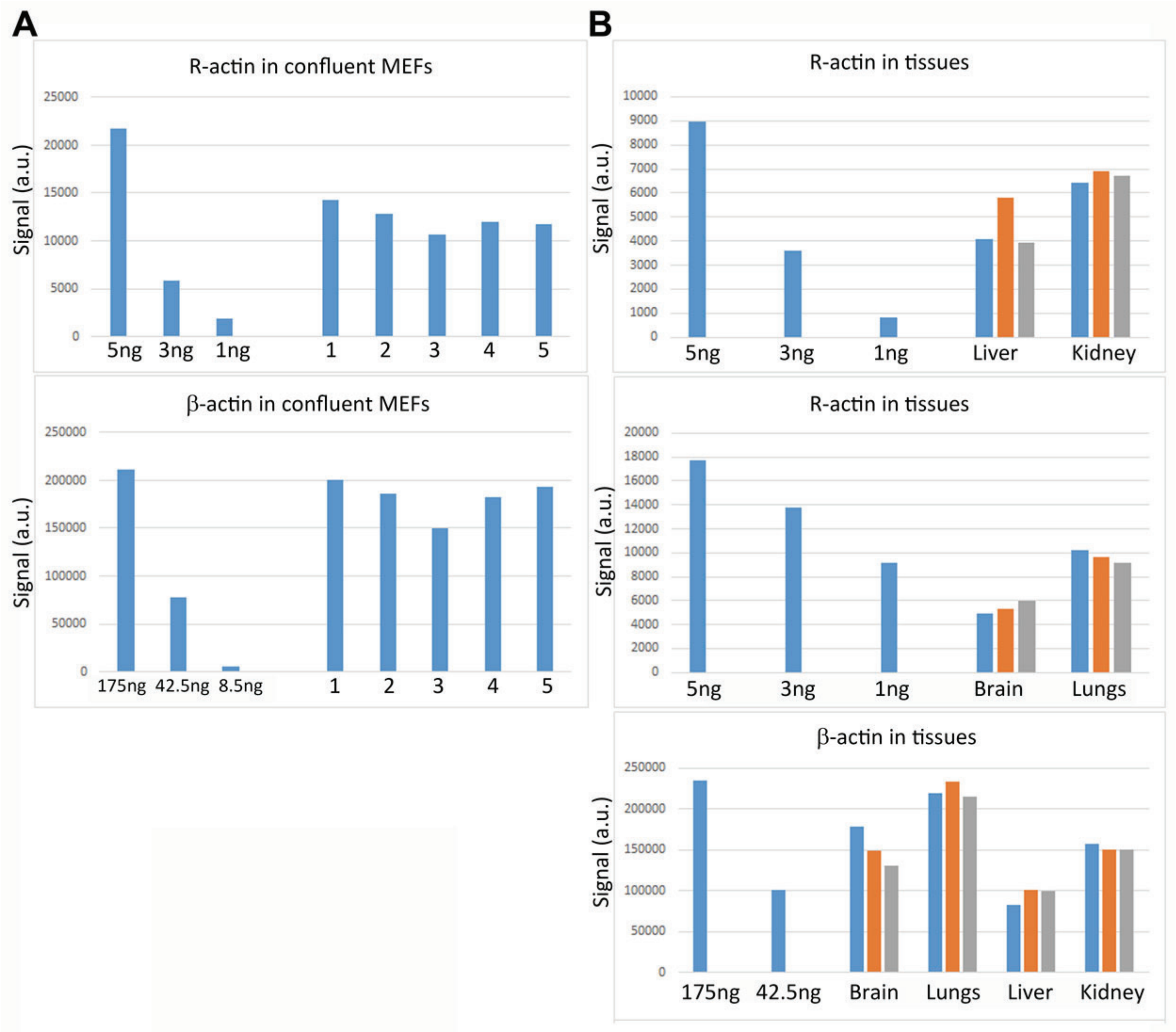

Figure S2. Raw data on the antibody signal for the quantifications used in Fig. 1B (A) and Fig, 4 (B).

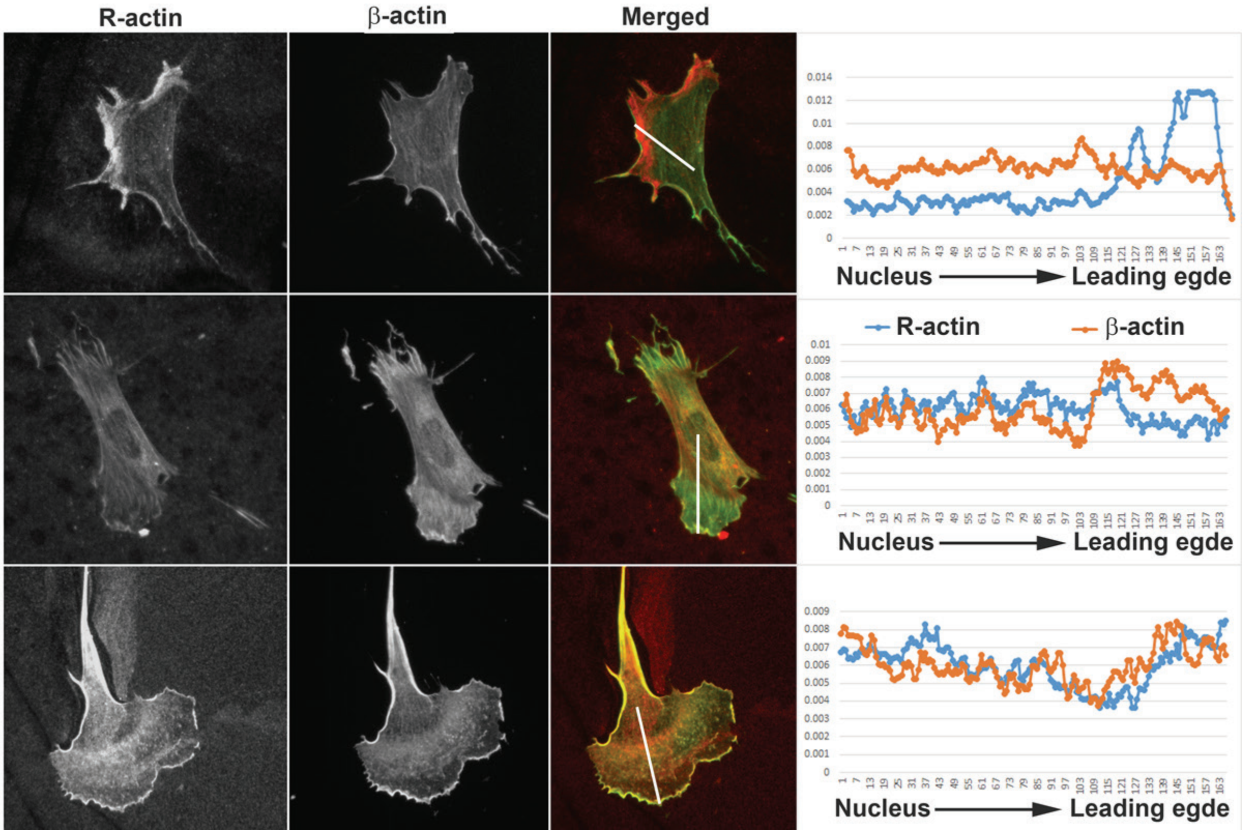

Figure S3. R-actin in cells exhibits local enrichment patterns that do not always coincide with the distribution of β-actin. Left, representative images of MEFs co-stained with R-actin and β-actin antibodies as shown. Right, intensity distribution of R-actin (blue) and β-actin (orange) along a line drawn from the cell center toward the periphery. Values on the y axis represent fractions of the total intensity along the line. Three representative cells with different R-actin and β-actin distribution patterns are shown.

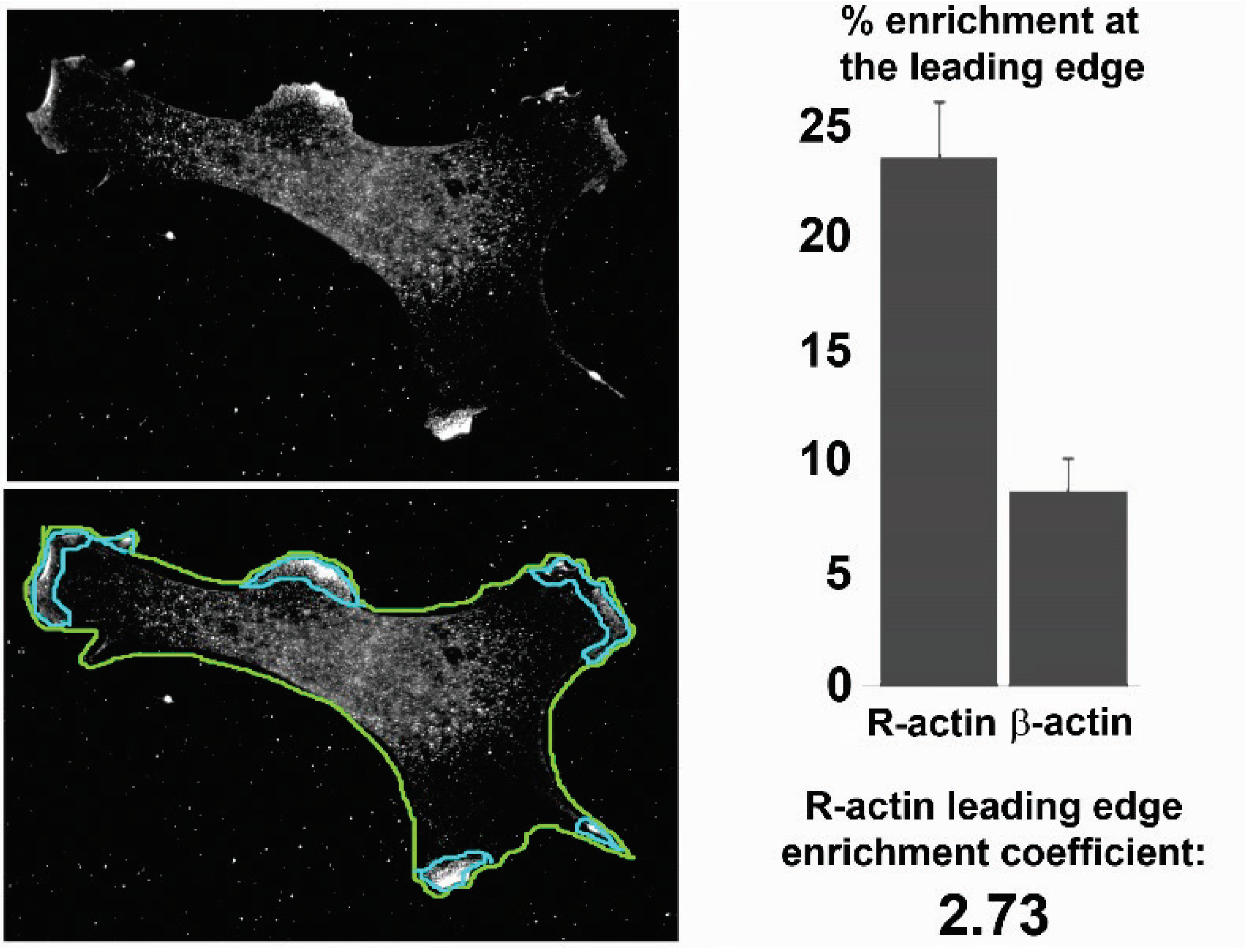

Figure S4. R-actin highest enrichment at the cell leading egde can account for nearly 25% of the total R-actin and exceed that of β-actin by nearly 3 fold. Left, representative images of a MEF cell stained with R-actin antibody. Top, raw image. Bottom, the same image with the outline of the whole cell (green) and R-actin-enriched leading edge (blue) used for quantification of R-actin fluorescence in the whole cell and its fraction at the leading edge, respectively. Right, percent enrichment of R-actin and β-actin at the cell leading edge, calculated from images similar for those shown on the left for R-actin, and cells independently stained with antibodies to β-actin (not shown). Five cells with the highest R-actin enrichment were used to calculate the "best case" percentage shown in the chart. Five cells of similar morphology were used to calculate the corresponding β-actin distribution. R-actin leading edge enrichment coefficient was calculated by dividing R-actin leading edge percentage (approximately 23%) by β-actin leading edge percentage (approximately 8%).
